# Cancer drug funding decisions in Scotland: impact of new end-of-life, orphan and ultra-orphan processes

**DOI:** 10.1186/s12913-017-2561-0

**Published:** 2017-08-30

**Authors:** Liz Morrell, Sarah Wordsworth, Howell Fu, Sian Rees, Richard Barker

**Affiliations:** 1 Oxford-UCL Centre for the Advancement of Sustainable Medical Innovation, Radcliffe Department of Medicine, University of Oxford, Room 4403, Level 4, John Radcliffe Hospital, Headley Way, Headington, Oxford, OX3 9DU UK; 2 Health Economics Research Centre, Nuffield Department of Population Health, University of Oxford, Old Road Campus, Roosevelt Drive, Headington, Oxford, OX3 7LF UK; 3 Oxford NIHR Biomedical Research Centre, University of Oxford, Oxford, UK; 4 Health Experiences Institute, Nuffield Department of Primary Care Health Sciences, University of Oxford, 23-38 Hythe Bridge Street, Oxford, OX1 2ET UK

**Keywords:** Scottish medicines consortium, NICE, End of life, Orphan, Rare, Cancer, Access, Funding, Cost-effectiveness

## Abstract

**Background:**

The Scottish Medicines Consortium evaluates new drugs for use in the National Health Service in Scotland. Reforms in 2014 to their evaluation process aimed to increase patient access to new drugs for end-of-life or rare conditions; the changes include additional steps in the process to gain further information from patients and clinicians, and for revised commercial agreements. This study examines the extent of any impact of the reforms on funding decisions.

**Method:**

Data on the Scottish Medicines Consortium’s funding decisions during 24 months post-reform were extracted from published Advice, for descriptive statistics and thematic analysis. Comparison data were extracted for the 24 months pre-reform. Data on decisions for England by the National Institute for Clinical and Health Excellence for the same drugs were extracted from published Technology Appraisals.

**Results:**

The new process was used by 90% (53/59) of cancer submissions. It is triggered if the initial advice is not to recommend, and this risk-of-rejection level is higher than in the pre-period. Thirty-eight cancer drugs obtained some level of funding through the new process, but there was no significant difference in the distribution of decision types compared to the pre-reform period. Thematic analysis of patient and clinician input showed no clear relationship between issues raised and funding decision. Differences between SMC’s and NICE’s definitions of End-of-Life did not fully explain differences in funding decisions.

**Conclusions:**

The Scottish Medicines Consortium’s reforms have allowed funding of up to 38 cancer drugs that might previously have been rejected. However, the contribution of specific elements of the reforms to the final decision is unclear. The process could be improved by increased transparency in how the non-quantitative inputs influence decisions. Some disparities in funding decisions between England and Scotland are likely to remain despite recent process convergence.

## Background

Drugs to treat rare conditions (including those formally defined as orphan and ultra-orphan drugs), and drugs to treat patients in their last months of life, have been the subject of contentious funding decisions in many countries, including the United Kingdom (UK). For rare conditions, small patient populations often result in the drugs being expensive, and they may not have the necessary level of evidence when submitting to reimbursement agencies to support a funding decision with certainty. For drugs related to end-of-life care (notably in cancer), there is a belief that extensions to the last months of life are particularly valuable to patients and their families [1] although the empirical data on public preferences for funding such drugs are equivocal [2].

Health Technology Appraisal is a devolved responsibility in the UK, and England and Scotland responded differently over time to the challenges of assessing these drugs. The first adjustment was by National Institute for Health and Care Excellence (NICE) in England in 2009, introducing its ‘End of Life Criteria’, which define situations in which evaluation committees can place greater weighting on health gain for drugs to treat patients with short life expectancy [3]. The Scottish Medicines Consortium (SMC) did not make similar adjustments at that time; however in 2012 ‘modifiers’ were introduced which allow SMC greater flexibility under specific conditions, in their acceptance of uncertainty in the economic case or a high cost per Quality-Adjusted Life Year (QALY); the uncertainty modifier applies to orphan drugs, but end of life is not specifically mentioned [4].

SMC introduced a further set of reforms in 2014, specifically for medicines to treat EOL and rare conditions. The changes take the form of an add-on process that is triggered only if the initial advice of SMC’s New Drug Committee (NDC) is not to recommend funding [5, 6] and include:

- Definitions of end of life, orphan, and ultra-orphan conditions. A drug may qualify under rarity, end-of-life, or both (for example a rare cancer).
- Introduction of a Patient And Clinician Engagement (PACE) meeting; purpose is to clarify aspects of value perceived by patients and clinicians that are not fully captured in the QALY, implicitly supporting acceptance of medicines with a cost per QALY above the standard threshold.
- Option for the submitting company to propose or modify a Patient Access Scheme (PAS) at the PACE stage.
- An additional framework of non-scored criteria for evaluating ultra-orphan drugs.

The SMC definitions are different from the end-of-life criteria used by NICE, both structurally and numerically (Table 1), and are more permissive than NICE’s current criteria.

**Table 1:** SMC and NICE criteria for consideration as an end-of-life or rare condition

NICE End-of-Life criteria	SMC End-of-Life and Rarity criteria
the treatment is indicated for patients with a short life expectancy, normally less than 24 months;	EoL: medicine to treat a condition at a stage that usually leads to death within 3 years.
and	
the treatment offers an extension to life, normally of at least an additional 3 months;	
and	or
for small patient populations normally not exceeding a cumulative total of 7000 for all licensed indications in England *(ie 1 in 7700*)*	Orphan: medicine to treat a condition affecting fewer than 2500 people in a population of 5 million) *(ie 1 in 2000).*
	or
	Ultra-orphan: medicine to treat a condition with a prevalence of 1 in 50,000 or less.

Sources: NICE [3] SMC [7]

* this criterion was removed in April 2016

The 2014 SMC changes are significant in the context of UK health technology appraisal, as a novel approach to incorporating broader aspects of value into cost-effectiveness analysis. With continued calls for NICE reform from advocacy groups, and similar pressures in other countries, it is important to evaluate the Scottish approach as a possible model for assessing value beyond the QALY, or as a pointer to key factors to include. Further, the changes could have important effects on patient access to new drugs in Scotland, with implications for spending priorities within NHS Scotland and for cross-border parity of patient access between the nations of the UK. Cancer is of particular interest, as a category that is expected to benefit from the end-of-life element, and where there are existing disparities in access arrangements, due to the existence of the Cancer Drugs Fund (CDF) in England and the pre-existence of NICE’s EOL criteria. Hence this paper evaluates the impact of the changes on funding decisions in the first 2 years of their operation, focusing on cancer, with the aim of contributing to ongoing debate on evolution of health technology assessment processes in the UK and elsewhere.

## Methods

SMC advice on submissions based on the new processes was published from October 2014 [6], so advice published from October 2014 to the date of analysis (September 2016) was selected. Comparison data for the 2-year pre-period were taken from advice published October 2012 to September 2014. Full and Abbreviated Submissions, Resubmissions, Superseded Advice and Independent Review Panels were included; non-submissions and Marketing Authorisation withdrawals were excluded. Resubmissions (ie re-application for a drug that had previously had a Not Recommended final decision from SMC) were analysed as part of the complete data set, and also separately, as we hypothesised they could be informative on any differences in decisions following the reforms. Cancer drugs were identified as British National Formulary (BNF) category 8: Malignant disease and immunosuppression, as classified by SMC, and selecting submissions for cancer indications.

Advice Summaries and Detailed Advice (pdf) documents were accessed from the SMC website, and data extracted and tabulated in Excel for descriptive statistics and thematic analysis. Data included submission type, EOL and rarity status, decision, incremental cost effectiveness ratios (ICERs), restrictions, use of PACE and PAS, and the PACE summaries. Descriptive statistics of the extracted data were analysed in Excel.

Because a simple before/after analysis does not isolate the effect of the changes, we took advantage of the structure of the reform, as an additional process triggered only for submissions that received a preliminary NDC position of a) Not Recommended, or b) Restricted more narrowly than requested by the manufacturer [7]. We therefore made the assumption that all drug candidates using the new process were initially Not Recommended, and that a change in that initial decision was due to use of the new process. Drugs using the new process were identified by the statement in the Advice headline that the submission was assessed “under the end of life/orphan/ultra-orphan process”.

Thematic analysis of the PACE meeting summaries was carried out by an inductive process: that is, by constructing themes suggested by review of the text rather than an imposed framework. Themes were developed by determining the core idea of each phrase, and linking similar ideas together to form themes. A given phrase was only linked to one theme. Related themes were grouped together for ease of presentation and understanding. The analysis was carried out by two researchers. One researcher developed an initial thematic framework from a sample of PACE summaries; a second researcher analysed the majority of the summaries, making adjustments to the framework as needed and in discussion with the first researcher, who completed the analysis. Thematic analysis was carried out using MaxQDA software (v12).

Data on NICE appraisals for the same drugs were accessed on the NICE website, and data extracted from the published Technology Appraisal Guidance documents (Final Appraisal Documents only; Appraisal Consultation Documents were excluded as these could change as a result of consultation). Data included approval status, whether EOL criteria were met, and reason for not meeting EOL criteria. For comparison of access between countries, restricted or optimised approvals were considered as ‘access’ along with full recommendation, and the most recent decision on a given drug-indication (that is, the one active in September 2016) was included to provide a point-in-time snapshot of drug availability.

Coverage by the Cancer Drugs Fund in England was determined from the CDF list of September 2015, and the relevant CDF Decision Summaries, accessed from the NHS England website.

## Results

### Submissions, resubmissions, and access

**Fig. 1:**
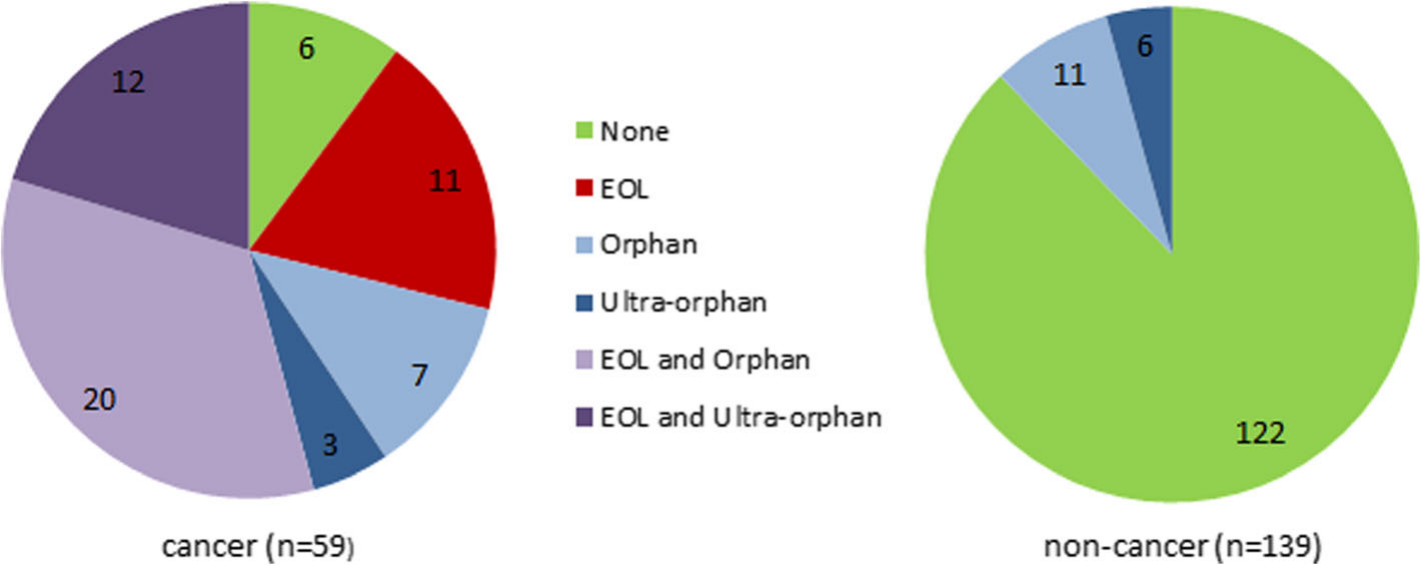
Use of criteria for new SMC process, cancer vs non-cancer Decisions October 2014–September 2016

Figure 1 shows the number of submissions that used the new process in the two-year period following its introduction. The process was used predominantly in cancer submissions, and most cancer submissions were made under at least one of the new criteria. All end-of-life submissions were cancer drugs. Apart from two cancer drugs and one non-cancer, all qualifying submissions made use of the PACE meeting, although those three drugs did have PAS. Note that Advice documents do not indicate the timing of the PAS, ie whether it was proposed at the later stage allowed by the new process, and for this reason use of PAS is not considered further in this analysis.

**Fig. 2:**
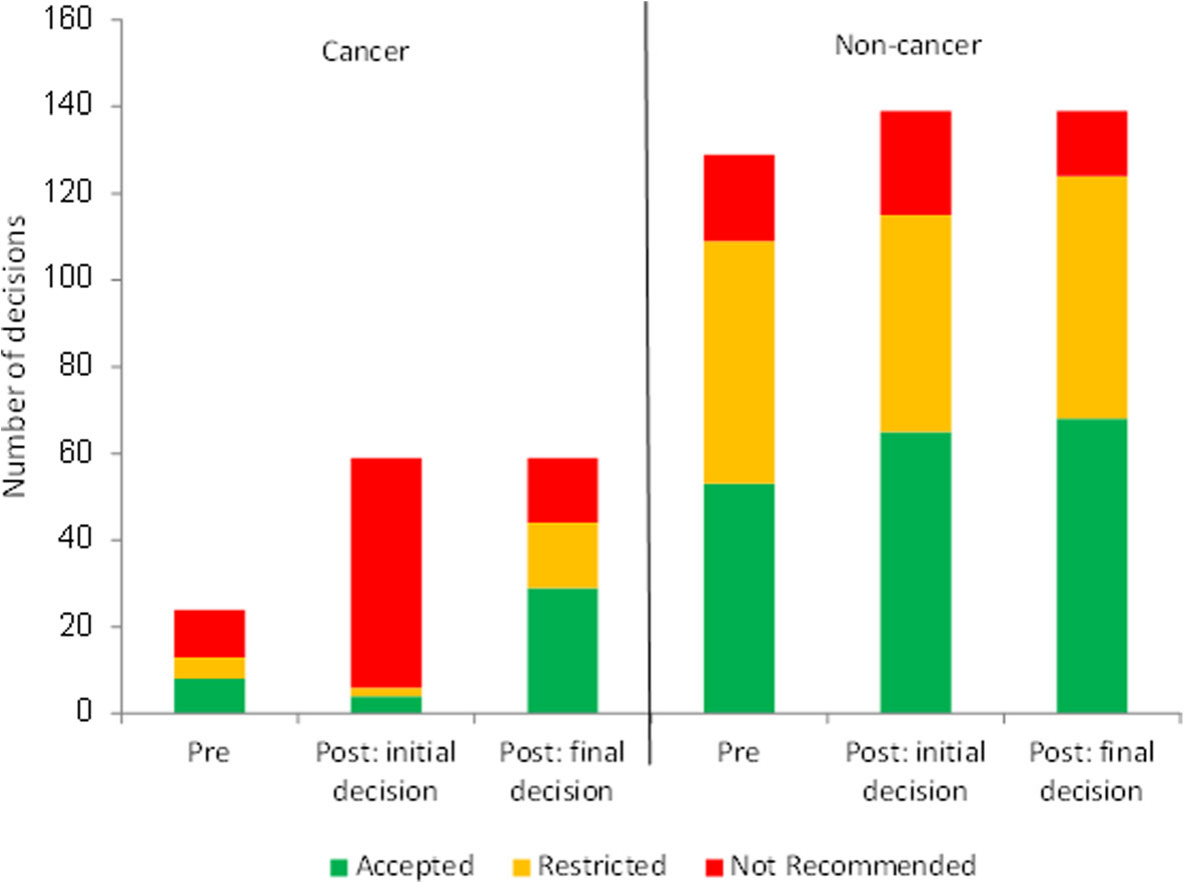
Distribution of SMC decisions pre-and post-reforms Pre: October 2012-September 2014 Post: October 2014-September 2016 Initial decision: distribution of initial NDC decisions Final decision: distribution of actual decisions, after use of the add-on process

Considering all submissions since the reforms compared to the period immediately before, the number of cancer submissions was higher post-reforms (Fig. 2). Although the proportion of cancer drugs approved for funding (Accepted or Restricted) increased from 54% in the pre-period to 75%, the change in proportions of the three decision types was not statistically significant (chi-squared test, *p* > 0.05).

In cancer, 53 of the 59 submissions (90%) were considered under the new process (Fig. 1), which we assume indicates they had an initial Not Recommended decision [6]. If these initial decisions had been confirmed, the resultant distribution of decisions would have been significantly different from the pre-period (‘Pre’ vs ‘Post: initial decision’: chi-squared test, *p* < 0.001). In the final decisions, however, only 15 of them were actually Not Recommended; if all 53 would have been Not Recommended under the original process, this represents 38 drugs that gained market access (Accepted or Restricted status) due to following the new process. Similarly, of the 17 non-cancer submissions assumed to be at risk of rejection, 9 gained some access.

There were 18 cancer resubmissions (ie drugs previously Not Recommended by SMC) post-reform, an increase on the six in the pre-period. Fifteen of the resubmissions used the new process, which as above we assume to indicate a preliminary Not Recommended decision, and 10 were subsequently funded (Accepted or Restrict status), as were all three that did not trigger the new process. Review of the Detailed Advice documents for the resubmitted drugs found that the resubmissions contain multiple changes compared to the original; the resubmissions typically refer to the same clinical data as the original submission, but might propose alternative cost effectiveness models, re-analyse for specific subgroups, or include confidential commercial arrangements. Hence it is not obvious whether each decision to fund a previously Not Recommended drug is due to the new process, or to other changes that improve the proposition such that the drug can now be approved.

### Impact of PACE meetings

A thematic analysis of the PACE meeting summaries in the SMC Advice is presented in Table 2, for the 45 unique cancer PACE meeting summaries. All PACE summaries commented on the benefit patients derived from the drug, and the effect on treatment pathways and drug choices, with most also commenting on at least one aspect of the specific condition. Some of the themes provide perspective on the value patients attach to the clinical benefits of the drugs: for example, extended overall survival translates into highly valued time with family, and improved progression-free survival means that patients remain out of treatment, and can continue with some level of normal life and activities. Similarly, having additional treatment options available provides hope for patients and families, and reduces anxiety about the future. There is no obvious relationship between the points covered and the funding decisions (chi-squared test, *p* > 0.05).

**Table 2:** Thematic analysis of cancer PACE meeting summaries

Themes	Frequency (*n* = 45)		Incorporated in CEE?
Characteristics of the disease:	40		
Poor prognosis		30	~
Heavily symptomatic		24	~
Devastating impact on patient and family		19	x
Patients are young		12	x
Psychological impact on patient		10	√
Patient benefits of the drug:	45		
Tolerability		31	~
Increase in overall survival		26	√
Improved progression-free survival		21	√
Issues with status quo treatments		19	√
Productivity		18	x
Value of extra time		17	√
Normal life		17	~
Better symptom control		16	√
Independence		16	~
Quality of life		15	√
Reduces anxiety/gives hope		11	~
Trial evidence underestimates drug benefit		10	√
Drug options and treatment pathways:	45		
Currently limited options		30	√
Convenience		23	x
Additional treatment option		14	~
Innovative therapy		13	x
No recent development in this disease		10	x
General issues:	27		
(Very) strong support for this medicine		14	x
Should be made available in line with indication		11	x
Total comments		527	

Includes themes mentioned in 10 or more PACE meeting summaries

√ Explicitly incorporated in cost-effectiveness evaluation (CEE); ~ Partially or indirectly incorporated; x Not incorporated

Among the high-frequency themes, although some are explicitly reflected in a typical cost effectiveness analysis (for example, overall survival, or symptom reduction), others are not, but may be considered during committee deliberations: for example, convenience for patients, impact on families, and hope. Some are partially or indirectly considered: for example, poor prognosis with an immediately shortened life expectancy would be covered by end-of-life considerations, but the severity of the condition per se is not formally weighted; similarly ‘independence’ is reflected to the extent it is symptom-dependent, and via the ‘usual activities’ and ‘self-care’ dimensions of EQ-5D, but not valued in its own right.

Eleven PACE meeting summaries commented on the need for the drug to be funded in its full licenced indication. These may be candidates where NDC was minded to impose a tighter restriction than requested by the manufacturer, although that information is not explicitly available.

### Comparison with England

To examine the impact of the differences between SMC and NICE criteria, we compared the post-reform evaluations by SMC with appraisals of the same drugs by NICE, considering separately the impact on the designation (whether the drug qualified under EOL or rarity) and the decision (whether the drug was recommended for funding). Note this analysis is based on a cohort of completed cancer appraisals from Scotland, so conclusions cannot be drawn regarding access or appraisal timings overall between the two countries.

**Fig. 3:**
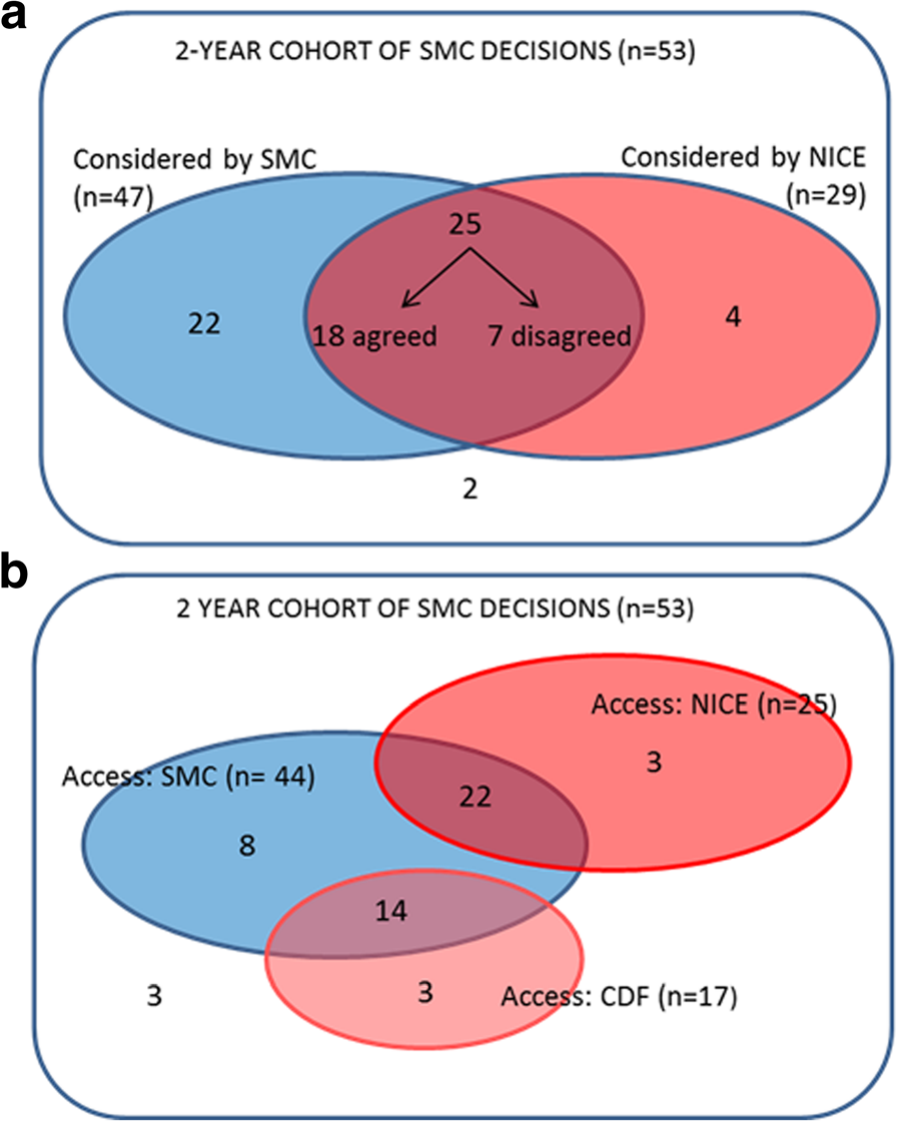
Comparison of SMC and NICE recommendations for EOL designation and access **a**. Comparison of EOL designation: number of submissions considered for granting of EOL/rare status by SMC (blue) and NICE (red), and level of agreement where both agencies gave a designation **b**. Comparison of access to drugs: number of drugs funded in Scotland (blue) and England (red), via SMC, NICE and CDF decisions

Comparison of the EOL designations shows that in the majority of cases (72%), the designation agreed between SMC and NICE despite the differences in the definitions. Of the active SMC cancer drug appraisals from the study period (*n* = 53), 25 had been considered for EOL designation by both agencies (Fig. 3a); in 16 of these cases both agencies conferred EOL status, and in two further cases, both agencies accepted a criterion allowing flexibility in assessment (EOL for NICE, rarity for SMC). Where a different designation was given, the reasons were the difference in definition of short life expectancy (4/7) and NICE’s requirement for a 3-month increase in survival benefit (3/7).

Nine cases had no EOL designation from one or both agencies (2 neither, 4 NICE only, 3 SMC only) because a decision to fund was reached without the need to trigger EOL consideration. Further reasons for absence of an EOL designation by NICE (ie designation by SMC only), are listed in Table 3; the majority are due to appraisals still pending at NICE.

**Table 3:** Reasons for SMC drugs having no NICE designation for EOL

	Number of submissions
No NICE appraisal:	
In development	10
Suspended	2
Non-submission by the company	2
Not scheduled for NICE appraisal	2
NICE agreed to fund without EOL consideration	3
NICE ICER too high regardless of EOL status	2
Appraised by NICE pre-2009 (ie before introduction of EOL criteria)	1
Total	22

Comparison of NICE and SMC decisions on this cohort of 53 cancer drugs identified 25 cases of differential recommendations between SMC and NICE in September 2016 (Fig. 3b), with 3 drugs funded only in England, and 22 only in Scotland. Of these, 10 are due to different decisions between SMC and NICE (ie SMC yes, NICE no), with the remaining 12 having no appraisal from NICE. Among the 10 divergent decisions, in 2 cases the drugs were classified as EOL by both agencies, so differences in decisions are not simply explained by EOL status. This is further illustrated by considering the 18 cases in Fig. 3a where the agencies agreed on EOL status: in 5 cases, different decisions were reached, with 3 funded by NICE only, and 2 by SMC only.

For a complete description of the differences in access between the countries from the patient’s perspective, we need to consider the totality of access arrangements, which includes the CDF in England. Because the CDF funds some drugs that are not available in Scotland, overall we identify 14 examples of differential access, with 8 drugs funded in Scotland only and 6 England only (Fig. 3b).

## Discussion

This analysis of funding decisions indicates that the SMC changes appear to have resulted in increased access to drugs for end of life and rare conditions, with up to 38 cancer drugs gaining access that they might not have had pre-reforms. The new, additional process was used most commonly for cancer drugs, and the proportion of cancer drugs triggering this process (due to a preliminary Not Recommended decision) is higher than would be predicted by the Not Recommended rate in the pre-period. The relative contribution of the PACE meeting, additional PAS opportunity, and increased ICER flexibility is unclear from this work. Differences between Scotland and England in classification of drugs as EOL lead to cross-border differences in EOL status, but do not fully explain differences in access; other factors include the agencies’ timeframe for appraisals, and which drugs are within their remit.

Our findings are consistent with early comments [8, 9] and a report [10] from SMC, and the findings of the recent Montgomery review [11], all of which suggest the changes are enabling SMC to approve drugs they could not have accepted before. They are also consistent with findings of Garau et al. [12] and Barham [13] in similar analyses. Both of those studies take a longer pre-period, creating a larger dataset, whilst this study chose 2 years to compare with the most recent submissions pre-reforms. Neither those studies, nor this one, have attempted a complete ICER analysis, because of the challenges of extracting comparable numbers from the appraisal documents, due to confidentiality and the presentation of ranges or multiple ICERs. To the best of our knowledge, our study is unique in providing the within-submission comparison, thematic analysis, and comparison with England.

The higher than expected proportion of the cancer submissions triggering the new process could be because this was an inherently challenging cohort of drugs, or because the reformed process – particularly the ICER flexibility – attracted submissions at higher ICERs than in the past. Alternatively, it may be that NDC’s readiness to use the new process is higher than the historical rate of Not Recommended decisions; for example, by being cautious in giving an Accept recommendation, which denies the opportunity for a new or revised PAS and a PACE meeting, thus losing an opportunity to gather additional information and potentially creating difficulties if SMC subsequently makes a Not Recommended decision. Irrespective of the reason, SMC appears to be handling more PACE meetings than might have been anticipated from previous decisions, and this may have implications for resources within SMC.

The nature of the impact of the PACE meetings on funding decisions remains unclear. Similarities between the PACE summaries may be created in part by the structured nature of the meeting and summary template, and the need to provide a concise summary for the appraisal committee, although a study published by Cancer52 found that PACE participants thought the summaries were an accurate reflection of the discussion [14]. The process could perhaps be improved by increased transparency in how the PACE input has been used. A subset of the themes are additional information that is not reflected directly in the quantitative cost-effectiveness analysis; of note, several of these are consistent with ideas for process reform considered by NICE under Value-Based Pricing (eg severity of the condition, innovation, wider social benefits) [15], whilst others such as the value of hope, and impact on the family, suggest further societal values that could be considered for future reform.

Our analysis identifies disparities in funding decisions between Scotland and England reflecting both process and decision differences between SMC and NICE. From a patient perspective, this disparity was reduced in reality by the existence of the CDF in England, which funded several of the drugs rejected or pending by NICE. The future funding of these CDF drugs is still being determined. The CDF reforms remedied some of the process differences between NICE and SMC, notably: a) removal of the rarity requirement in NICE’s end-of-life criteria, bringing the definitions into closer alignment; b) expectation that NICE will publish a decision more quickly than previously, normally within 90 days of the marketing authorisation for cancer drugs; and c) requirement that all new cancer drugs will be referred to NICE, which was previously not the case. However, as we saw in this cohort of drugs, even where both agencies have completed an evaluation under EOL conditions they can still come to different decisions, so some differences in access are likely to remain, reflecting other process differences, differences in clinical practice, implicit preferences, and varying commercial arrangements with the pharmaceutical industry.

A limitation of our study is our assumption that all the submissions that triggered the new process, would have been rejected had the reforms not been in place. Some of them might in fact have been granted some level of access following further deliberation and negotiation. Importantly, NDC do not use the ‘modifiers’ available to SMC to allow additional flexibility in specified circumstances; application of these modifiers may have supported a Recommended decision by SMC even without the reforms, despite an initial Not Recommended position from NDC. Comments from stakeholders on the NDC decision, or new analysis suggested by the NDC review, could also lead to SMC reversing an initial Not Recommended NDC position. In either case, our analysis would overestimate the number of additional approvals permitted by the reforms. Further, as outlined in the Methods section, the new process can also be triggered if the NDC are minded to impose a tighter restriction on use than proposed in the submission; however, if we take account of the 11 cases identified in the PACE analysis - assuming they were at risk of involuntary restriction rather than rejection - our observations on distribution of decisions still hold.

A second limitation is our use of information available in the public domain, which includes summaries by SMC staff and excludes commercially confidential information, leading to risk of bias due to missing and secondary information - although our analysis does reflect the summary information available to SMC committee at the point of decision. The summarised nature of the public documents in particular limits our ability to determine which elements of post-reform resubmissions – the new process, or other improvements to the case for funding – led to the reversal of the previous decision. Further, we have not considered whether the additional spending represents value for money for NHS Scotland, which would require a comparison of the health gain achieved with expenditure; estimating the actual costs is particularly challenging given the confidentiality of any commercial arrangements. If acceptance of these drugs for funding is at higher ICERs than would be accepted for other drugs, opportunity costs are imposed elsewhere in the health system. It is not clear from the empirical literature that such prioritisation of medicines for end of life and rare conditions is in line with the preferences of the Scottish public [16].

Strengthening the PACE analysis by using verbatim transcripts of the meetings rather than summaries, and supplementing with qualitative work exploring the experiences of key players in the process, could provide additional insight into the impact of PACE on decisions. In addition, the reforms occurred in parallel with SMC moving to holding appraisal committee meetings in public, and increase of the New Medicines Fund to £90million to help cover the cost of these medicines. In combination, these changes may have had additional effects beyond numbers of approvals, such as affecting buy-in to decisions, or encouraging pricing flexibility, and the contribution of these diverse elements deserves additional exploration. Further work is also needed to determine whether the choices made under the new SMC process provided value for money, ideally by examining actual outcomes from use of the drugs in practice. The CDF in England has been criticised for failing to provide such an evaluation of its expenditure [17], and data are currently not available consistently across Scotland [11]; findings from usage in Scotland may provide useful insight for future evolution of technology evaluation processes and potentially for increased unification of decisions across the UK.

## Conclusion

We identified up to 38 cancer drugs that were funded post-reform that might previously have been rejected, but the contribution of each element of the reforms to the funding decision remains unclear. Differences in decisions between England and Scotland are not fully explained by differences in definitions of EOL/rare conditions, and disparities in access are likely to remain despite recent changes in England. Future improvements to the process could include enhanced transparency in use of the PACE input, and evaluation of patient outcomes from funding access to these drugs.

## Abbreviations

BNF: British national formulary
CDF: Cancer drug fund
EOL: End of life
ICER: Incremental cost effectiveness ratio
NDC: New Drugs Committee
NHS: National health service
NICE: National institute of health and clinical excellence
PACE: Patient and clinician engagement
PAS: Patient access scheme
QALY: Quality-adjusted life year
QoL: Quality of life
SMC: Scottish Medicines Consortium
UK: United Kingdom
